# Effect of indigestible dietary protein on growth performance and health status of weaned pigs

**DOI:** 10.1093/jas/skaf372

**Published:** 2025-10-25

**Authors:** Taiwo J Erinle, Marllon J K de Oliveira, John K Htoo, S Maria Mendoza, Daniel A Columbus

**Affiliations:** Prairie Swine Centre, Inc., Saskatoon, SK S7H 5N9, Canada; Department of Animal and Poultry Science, University of Saskatchewan, Saskatoon, SK S7N 5A8, Canada; Prairie Swine Centre, Inc., Saskatoon, SK S7H 5N9, Canada; Evonik Operations GmbH, Hanau-Wolfgang 63457, Germany; Evonik Corporation, Kennesaw, GA 30144; Prairie Swine Centre, Inc., Saskatoon, SK S7H 5N9, Canada; Department of Animal and Poultry Science, University of Saskatchewan, Saskatoon, SK S7N 5A8, Canada

**Keywords:** acute-phase protein, antioxidant status, diarrhea severity, inflammatory biomarkers, microbial proteolytic fermentation metabolites, undigested dietary protein

## Abstract

Feeding diets containing excess dietary protein (DP) is associated with increased hindgut protein fermentation, often resulting in post-weaning diarrhea. However, the response to DP content has been variable in piglets. The indigestible dietary protein (IDP) content of diets may be more related to negative health and performance outcomes than total DP. The objective of this study was to evaluate the impact of diet IDP content on performance, immune response, and fecal consistency score (FCS) in newly weaned pigs. Eighty mixed-sex weaned pigs with an average initial body weight of 8.1 ± 0.31 kg were housed in groups of 5 pigs/pen and randomly assigned to 1 of 2 dietary treatments in a completely randomized design (*n* = 8 pens/treatment) for 28 d. Diets were formulated to contain similar total crude protein (22% [phase I] and 20% [phase II]), net energy, and to meet or exceed all other nutrient requirements, but differ in IDP content (low IDP, 2.74% [LIDP] or high IDP, 4.2% [HIDP]). During the first 7 d, FCS was visually scored, followed by a weekly FCS and growth performance measurements. On d 9 post-weaning, blood, digesta from ileum, cecum, and colon, and fecal samples were collected in the morning from 1 pig/pen without prior fasting. Additional blood was also collected from 1 pig/pen on d 28. There was no effect of IDP on feed intake (*P* > 0.10). Low IDP increased average daily gain by 7% during phase II and 4% during the overall period compared to HIDP (*P *< 0.05). Gain:feed was lower and higher in LIDP-fed pigs during phases 1 and 2, respectively, compared to HIDP (*P *< 0.05). Pigs fed LIDP diet had improved FCS on d 6 and overall compared to HIDP (*P *< 0.01). Plasma superoxide dismutase concentration was higher in LIDP-fed pigs compared to HIDP (*P *< 0.05). There was neither IDP, Day, nor their interaction effect on serum albumin, *interleukin* (*IL*)-6, *IL*-1β, and plasma alkaline phosphatase (*P *> 0.10). Oxidized glutathione (GSSG) was higher on d 28 in LIDP-fed pigs compared to d 9 (*P *< 0.05); but GSSG in pigs fed HIDP on both days. In the cecum, LIDP increased the concentrations of cadaverine, spermine, allantoin, creatinine, and *N*1-acetylspermidine (*P *< 0.05) and reduced phenylethylamine, tryptamine, and choline (*P *< 0.05). High IDP increased valerate concentration in the colonic digesta (*P *< 0.05). The results indicate that feeding LIDP diet improved growth performance and oxidative status, reduced diarrhea severity, while increasing some biogenic amines in nursery pigs.

## Teaser

Given the transition from highly digestible sow’s milk to less digestible plant-based protein diets, improving weaning outcomes may be achieved by feeding low indigestible protein diets, rather than just reducing total dietary protein content. The findings of the present study provide better insights into nutritional management, via formulating based on indigestible protein, for improving performance and gut health of newly weaned pigs.

## Introduction

At weaning, piglets are suddenly switched from highly digestible sow’s milk to less digestible plant-based diets. Disruption of gut development (e.g., reduced nutrient availability and disease challenge) during this time can have significant impact on gut function, including digestive capacity and intestinal barrier function (Liu et al., 2022). Therefore, the composition of diets is critical for maintaining piglet growth and gut health, and nursery diets are largely formulated to contain highly digestible feed materials.

While protein remains an indispensable nutrient in swine nutrition, its source, quality (e.g., amino acid profile), and digestibility are pivotal in optimizing the performance and health of pigs. In contrast to plant-protein sources, animal-based diets are more digestible, which consequently improves voluntary feed intake, growth, and intestinal morphology of weaning pigs (Yun et al., 2005; Deng et al., 2023). For cost-efficiency, typical piglet diets contain varying protein sources, mainly plants, and could increase the amount of undigested protein reaching the hindgut, where it is subjected to proteolytic fermentation. Depending on the luminal concentration, metabolites of protein fermentation could distort hindgut homeostasis (Li et al., 2019a, 2019b; Zhang et al., 2020) , mucin production (Sittipo et al., 2019), intestinal epithelial cell formation (Gaskins, 2000), and promote diarrhea incidence in weaning pigs (Htoo et al., 2007).

Due to the perceived negative effects of high dietary protein (DP), efforts have been made to reduce DP in diets for weaned pigs while maintaining performance (Htoo et al., 2007). While a low-protein diet could reasonably be correlated to low diarrhea incidence (Kim et al., 2023), literature evidence on the DP reduction presents conflicting outlooks. For example, notwithstanding the addition of amino acids, a decrease in DP levels from 21% to 17% (Opapeju et al., 2008) and 19% to 14% (Lynegaard et al., 2021) was reported to reduce performance and diarrhea incidence in piglets. Contrary to low DP, increasing DP from 18.1% to 24% (Diether et al., 2024) and 22.63% to 18.26% (Fang et al., 2019) was shown to improve growth performance without causing diarrhea prevalence. In other studies, increasing DP from 17% to 24% (Pearce et al., 2024) or 16% to 20% (Rodrigues et al., 2021) did not affect growth performance or diarrhea incidence in pigs. Besides the total DP levels, an additional dietary factor with more profound underlying impacts might be a more relevant explanatory factor responsible for the inconsistencies across DP studies in weaning pigs.

In a recent meta-analysis by de Oliveira et al. (2025), increasing inclusion levels of plant-based protein sources in diets for weaning pigs significantly increased the amount of indigestible dietary protein (IDP) content, which has detrimental effects on the performance response of pigs. While this has yet to be demonstrated in vivo, it is believed that feeds with a high IDP index may increase the amount of protein available for proteolytic fermentation in the distal gastrointestinal tract (GIT) and consequently compromise weaning outcomes. The objective of the present study was to determine whether varying IDP levels would negatively impact growth performance, antioxidants and inflammatory biomarkers in blood, hindgut fermentation metabolites, and diarrhea severity of nursery pigs. It was hypothesized that a high IDP diet would negatively affect growth performance and immune status, increase hindgut fermentation metabolites, and promote diarrhea severity in newly weaned pigs.

## Materials and Methods

### Ethics statement

The experimental protocol (#20220082) was approved by the University of Saskatchewan’s Animal Research Ethics Board. The pigs were handled following the established guidelines by the Canadian Council on Animal Care (CCAC 2009).

### Animals, housing, diets, and experimental design

A total of 80 mixed-sex nursery pigs (Camborough Plus × C3378; PIC Canada Limited; 28 ± 2 d old; 8.1 ± 0.31 kg body weight [BW]) were obtained and used for this study at the Prairie Swine Centre, Inc. (Saskatoon, SK, Canada). Pigs were housed in pens, with 5 pigs/pen, balanced for BW in a temperature-controlled room (26 ± 2 °C). Pigs were randomly assigned to 1 of 2 dietary treatments over 2 blocks (n = 8 pens/treatment) in a randomized complete block design for 28 d. Dietary treatments (Tables 1 and 2) contained similar DP content (21%) but either a low IDP (2.74%, LIDP) or high IDP (4.20%, HIDP). The IDP levels were estimated as follows:


IDP (%)=Total DP (%) – Standardized ileal digestible DP (%)

**Table 1.** Ingredient and nutrient compositions of experimental diets (as-fed basis)^1^

	Diets^1^			
	7–11 kg		11–25 kg	
Ingredients, %	LIDP	HIDP	LIDP	HIDP
Corn	59.8	36.5	73.6	50.3
Soybean meal	9.61	9.61	9.61	9.61
Soybean protein concentrate	15.6	3.76	12.4	–
Corn DDGS	–	19.5	–	20.0
Whey permeate	10.0	10.0	–	–
Canola oil	0.56	4.46	0.42	4.32
Canola meal	–	12.0	–	12.0
L-Lysine HCl	0.50	0.76	0.47	0.75
DL-Methionine	0.29	0.23	0.23	0.17
L-Threonine	0.21	0.24	0.18	0.22
L-Tryptophan	0.10	0.12	0.09	0.12
L-Isoleucine	0.13	0.07	0.12	0.07
L-Valine	0.13	0.13	0.12	0.11
Limestone	1.30	1.41	1.21	1.33
Monocalcium phosphate	1.27	0.71	1.07	0.50
Salt	0.40	0.40	0.40	0.40
Vitamin-mineral premix^2^	0.15	0.15	0.15	0.15
**Calculated nutrient content^3^**				
Dry matter, %	89.1	87.0	87.9	85.8
Metabolizable energy, kcal/kg	3,340	3,354	3,323	3,333
Net energy, kcal/kg	2,500	2,500	2,500	2,500
Crude protein, %	21.0	22.0	19.7	20.5
SID crude protein, %	18.3	17.8	17.0	16.3
IDP, %	2.74	4.20	2.74	4.20
Total DF, %	10.4	15.7	10.8	16.2
Insoluble DF, %	9.63	14.9	10.1	15.4
Soluble DF, %	0.72	0.85	0.67	0.79
Insoluble DF: Soluble DF	13.4	17.5	15.2	19.5
Soluble DF: Total DF, %	0.07	0.05	0.06	0.05
Insoluble DF: Total DF, %	0.93	0.95	0.94	0.95
Calcium,%	0.80	0.80	0.70	0.70
Phosphorus, %	0.61	0.68	0.55	0.61
STTD phosphorus, %	0.40	0.40	0.33	0.33
Calcium: STTD Phosphorus	2.00	2.00	2.12	2.12
**Amino acids, % SID**				
Arginine	1.23	1.04	1.14	0.92
Histidine	0.46	0.45	0.44	0.42
Isoleucine	0.89	0.74	0.83	0.68
Leucine	1.55	1.57	1.53	1.53
Lysine	1.35	1.35	1.23	1.23
Methionine	0.55	0.52	0.49	0.46
Cysteine	0.26	0.29	0.25	0.28
Methionine + Cysteine	0.81	0.81	0.74	0.74
Phenylalanine	0.89	0.81	0.85	0.76
Tyrosine	0.58	0.52	0.54	0.48
Phenylalanine + Tyrosine	1.47	1.34	1.38	1.23
Threonine	0.85	0.85	0.78	0.78
Tryptophan	0.30	0.30	0.27	0.27
Valine	0.96	0.92	0.91	0.84

DF, dietary fiber; IDP, indigestible dietary protein; SID, standardized ileal digestible; STTD, standardized total tract digestible

1 Experimental diets with low IDP (LIDP) and high IDP (HIDP)

2 Supplied per kg of complete diet: vitamin A, 6,000 IU; vitamin D, 9.3 mg; vitamin E, 35 IU; menadione, 2.5 mg; vitamin B12, 0.02 mg; thiamine, 1.00 mg; biotin, 0.10 mg; niacin, 20 mg; riboflavin, 4 mg; pantothenate, 12 mg; folic acid, 0.50 mg; pyridoxine, 5.0 mg; Fe, 75 mg; Zn, 75 mg; Mg, 20 mg; Cu, 10 mg; Se, 0.15 mg, and I, 0.50 mg.

3 Nutrient content of diets based on estimated nutrient contents of ingredients according to NRC (2012) and analyzed AA content by AMINOLab of Evonik Operations GmbH.

**Table 2.** Analyzed nutrient content of experimental diets (as-fed basis)

	Diets^1^			
	7–11 kg		11–25 kg	
Item	LIDP	HIDP	LIDP	HIDP
Dry matter, %	90.3	89.9	88.8	89.5
Gross energy, kcal/kg	3,988	4,157	3,940	4,208
Crude protein %	21.7	22.3	20.0	20.8
**Total amino acids^2^**				
Methionine	0.50 (0.58)	0.56 (0.57)	0.51 (0. 52)	0.51 (0.51)
Cystine	0.32 (0.31)	0.38 (0.37)	0.33 (0.30)	0.37 (0.36)
Methionine + Cystine	0.83 (0.89)	0.94 (0.94)	0.84 (0.82)	0.88 (0.87)
Lysine	1.32 (1.46)	1.62 (1.54)	1.30 (1.34)	1.25 (1.42)
Threonine	0.97 (0.96)	1.06 (1.02)	0.92 (0.89)	0.97 (0.95)
Tryptophan	0.33 (0.33)	0.37 (0.34)	0.31 (0.30)	0.31 (0.31)
Arginine	1.32 (1.30)	1.27 (1.17)	1.28 (1.20)	1.09 (1.05)
Isoleucine	0.97 (0.97)	0.95 (0.87)	0.97 (0.91)	0.84 (0.81)
Leucine	1.82 (1.72)	1.89 (1.83)	1.85 (1.70)	1.94 (1.80)
Valine	1.09 (1.07)	1.17 (1.10)	1.09 (1.01)	1.26 (1.02)
Histidine	0.54 (0.52)	0.56 (0.54)	0.54 (0.49)	0.53 (0.51)
Phenylalanine	1.03 (0.98)	1.01 (0.96)	1.01 (0.94)	0.94 (0.90)
Glycine	0.82	0.93	0.82	0.84
Serine	0.97	1.00	0.98	0.95
Proline	1.13	1.40	1.27	1.42
Alanine	0.99	1.14	1.08	1.18
Aspartic acid	1.99	1.84	1.95	1.56
Glutamic acid	3.39	3.74	3.54	3.48

IDP, indigestible dietary protein; SID, standardized ileal digestible

1 Experimental diets with low IDP (LIDP) and high IDP (HIDP)

2 Analyzed values of individual amino acids with corresponding calculated values in brackets.

The two IDP levels were achieved using protein sources that were digestible (soy protein concentrate) and less digestible (canola meal and corn distiller dried grain with solubles). Corn-soybean-based crumble diets were formulated using the reported nutrient content and analyzed amino acids (**AA**) content of ingredients to meet or exceed nutrient requirements in two phases for 7–11 and 11–25 kg pigs according to NRC (2012) and AMINODat 6.0 (Evonik, 2021) recommendations using published nutrient content of ingredients according to NRC (2012) and AMINODat 6.0 . Pigs were allowed unrestricted access to feed and water.

### Growth performance

Pigs were individually weighed and per pen feed intake was determined on a weekly basis. The average pen BW and feed intake were used to determine the average daily gain (ADG), average daily feed intake (ADFI), and feed efficiency (gain:feed [G:F]) of pigs.

### Fecal consistency score

On d 0, 1, 2, 3, 4, 5, 6, 7, 14, 21, and 28, fecal samples were collected from individual pigs per pen via rectal palpation and visually scored for fecal consistency (FCS) following a metric adapted from Wellock et al. (2008). Briefly, a score of 1 was assigned to normal (solid/firm) feces, a score of 2 to pasty (semi-solid) feces, a score of 3 to moderately fluid (loose) feces, and a score of 4 to highly fluid (watery) feces. An FCS >2 is considered diarrheic.

### Sample collection

On d 9, one pig per pen with average BW was humanely euthanized sample collection using a non-penetrative captive bolt. Following euthanasia, blood samples were immediately collected via cardiac puncture into 10 mL sodium-heparin coated vacutainers (BD, Mississauga, ON, Canada) and anticoagulant-free vacutainers for collection of plasma and serum, respectively. Blood collected in vacutainers with no anticoagulant were allowed to clot. Subsequently, all blood samples were centrifuged at 2,500×*g* for 15 min at 4°C to obtain plasma and serum. The resulting plasma and serum supernatant were aliquoted into 2 mL microtubes and stored at −20°C until further analyses. Digesta samples were obtained from the distal ileum and mid-points of cecum and colon and fecal samples were collected directly from the rectum. Digesta and fecal samples were placed into 15 mL Falcon tubes and immediately frozen at –80°C until further analysis.

On d 28, blood samples were collected via jugular venipuncture and fecal samples collected via rectal palpation on one pig per pen representing the average BW. Samples were processed as described for d 9.

### Fecal and blood analyses

All fecal samples were analyzed for myeloperoxidase (MPO) activity (ab105136; Abcam, Cambridge, MA) following the manufacturer’s instructions and the sample processing procedure of Bloomer (2018). In brief, fecal samples were allowed to thaw at room temperature, immediately transferred and thoroughly mixed on ice, and subsampled into 2 mL microtubes. The fecal samples were diluted 1:1 with d.d. H_2_O, centrifuged at 6,000×*g* for 20 min at 4°C, and 750 µL of the resultant supernatant was transferred into 2 mL microtubes and further centrifuged at 7,000 × g for 10 min at 4°C.

Serum samples were analyzed for serum albumin (ALB) was analyzed by bromocresol green method using Chemistry Analyzer/Single Chemistry (Prairie Diagnostic Services, Saskatoon, SK, Canada), interleukin-6 (IL-6, ab100755; Abcam, Cambridge, MA), and interleukin-1β (IL-1β, ab100756; Abcam) according to manufacturers’ instructions. Plasma content of haptoglobin (ab205091; Abcam), alkaline phosphatase (ALP, DALP-250; Gentaur, Bioassay Systems, Hayward, CA, USA), superoxide dismutase (SOD; Item No: 706002, Cayman Chemical, Ann Arbor MI), reduced glutathione:oxidized glutathione (GSH:GSSG, ab138881; Abcam) were analyzed following the manufacturer’s instructions of the respective kits. For colorimetric or fluorometric assays, absorbances were measured using a colorimetric plate reader (EPOCH2C; BioTek Instruments, Inc., USA) or fluorometric plate reader (S1LF; BioTek Instruments, Inc., USA), respectively.

### Digesta collection and analysis

The ammonia nitrogen (NH_3_-N) concentration in ileal, cecal, and colonic digesta was measured spectrophotometrically based on the reaction of ammonium ions with sodium phenate, sodium nitroprusside, and hypochlorite reagents (Rhine et al., 1998), following a procedure previously described by Erinle et al. (2025). Briefly, colonic digesta samples were diluted with double-distilled H_2_0 at a ratio 1:1. Approximately 1 g of ileal, cecal, and diluted colonic digesta were centrifuged at 14,000×*g* for 10 min, and 20 µL of supernatant was transferred into glass tubes and covered with parafilm to prevent absorption of ammonia from the air. The standard solution was prepared by dissolving 0.4176 g ammonium sulfate (previously dried at 60 °C for 2 h) in 1 L of deionized water. Seven gradient levels of standard solution (0, 10, 20, 30, 40, 50, and 60 μL) were added into glass tubes for standard curve generation. Subsequently, 2 mL of sodium phenate, 3 mL of 0.01% sodium nitroprusside, and 3 mL of 0.02 N hypochlorite working solution were added into each tube containing standard or samples, covered with parafilm and incubated on a shaker for 1 h in the dark for color development. The absorbance was determined at 600 nm using a spectrophotometer (Model# 4001/4, Genesys 20; Thermo Scientific, USA). The concentration of volatile fatty acids (VFA) profile, short branched-chain fatty acids (BCFA), biogenic amines and precursors, and non-biogenic amines were analyzed using targeted quantitative metabolomics using reverse-phase LC-MS/MS on an ABSCiex4000 QTrap mass spectrometer (Applied Biosystems/MDS Sciex; Toronto, ON, Canada; Foroutan et al., 2019, 2020) and was performed at The Metabolomics Innovation Centre (Edmonton, AB, Canada).

### Statistical analysis

Datasets were verified for normality of error terms using the PROC UNIVARIATE model and the Shapiro-Wilk test in SAS 9.4 (SAS Institute, Inc., Cary, NC, USA) and outliers were identified and removed where the residual of error terms was ±3 standard deviation from the mean. Where normality could not be achieved, particularly in VFA and other fermentation metabolites, datasets were transformed using logarithm, square root, or cube root transformation functions, and the resultant means and their standard error of mean were back-transformed accordingly. For growth performance and FCS, pen was considered the experimental unit, while individual pig was the experimental unit for other parameters. The model analyzed FCS as repeated measures in a randomized complete block design (PROC GLIMMIX), while fecal MPO and blood parameters as repeated measure in the same design (PROC MIXED), including (1) IDP, (2) day, and (3) their interaction, as presented below:


$$\Upsilon_{\mathrm{ijk}}=\mu +\rho_{i}+\alpha_{j}+\beta_{k}+\alpha \beta_{jk}+\varepsilon_{\mathrm{ijk}},$$

where Υ_*ijk*_ = dependent variable; μ = the overall mean; ρ_*i*_ = random effect of block; α_*j*_ = the effect of IDP; β_*k*_ = the Day effect; αβ_*jk*_ = the interaction effect of IDP and Day; and ε_*ijk*_ = experimental error.

The repeated measure analysis was performed using an appropriate co-variance structure test with the lowest Akaike information criterion and Bayesian information criterion values. These main effects and their interactions were considered fixed, while block was considered random. Mean separation was determined using the Tukey-Kramer test with a significant level set at *P *< 0.05. A tendency towards significance was considered if 0.05 < *P *< 0.10. To have an overview of the changes induced by IDP on redox biomarkers and acute-phase protein in blood and fecal MPO (d 9) and the addition of growth performance (d 28), principal component analysis was performed using PROC PRINCOMP model of SAS 9.4. An eigenvalue >1 was used to identify the number of principal components (PC) contributing to total variance and, therefore, constitute the components used as factors. Component loading of variables was established to determine specific variables that were strongly correlated with retained principal components using the largest absolute eigenvectors in retained factors.

## Results

### Growth performance

Growth performance results of pigs fed LIDP or HIDP are shown in Table 3. Final BW was not affected by IDP content (*P *> 0.10). There was no IDP effect on weekly, phase, or overall ADFI of pigs (*P *> 0.10). Up to d 21, there was no IDP effect on ADG of pigs (*P *> 0.10). However, on d 28, during phase 2 (d 15–28), and overall (d 0–28), ADG was higher in pigs fed LIDP compared to HIDP (*P *< 0.05). Pigs fed LIDP had lower and higher G:F ratios during phases 1 (d 0–14) and 2 (d 15–28), respectively, compared to those fed HIDP (*P *< 0.05); however, overall G:F was not affected by IDP (*P *> 0.10).

**Table 3.** Growth performance of piglets fed low or high indigestible dietary protein

	Diets^1^			*P*-value
Item	LIDP	HIDP	SEM	IDP
Initial BW, kg	8.28	8.25	0.229	0.649
Final BW, kg	20.43	19.92	0.602	0.320
**Average daily feed intake, kg/d **				
d 0–7	0.23	0.21	0.014	0.100
d 8–14	0.50	0.50	0.018	0.871
d 15–21	0.73	0.73	0.027	0.832
d 22–28	1.10	1.08	0.033	0.715
Phase 1 (d 0–14)	0.36	0.36	0.006	1.000
Phase 2 (d 15–28)	0.92	0.91	0.022	0.718
Overall (d 0–28)	0.64	0.65	0.013	0.836
**Average daily gain, kg/d **				
d 0–7	0.18	0.16	0.018	0.489
d 8–14	0.39	0.41	0.026	0.630
d 15–21	0.54	0.47	0.037	0.114
d 22–28	0.76	0.66	0.019	0.004
Phase 1 (d 0–14)	0.24	0.27	0.015	0.113
Phase 2 (d 15–28)	0.64	0.57	0.036	0.048
Overall (d 0–28)	0.47	0.43	0.010	0.016
**Gain:feed, kg/kg **				
d 0–7	0.62	0.59	0.056	0.247
d 8–14	0.63	0.82	0.063	0.001
d 15–21	0.69	0.64	0.059	0.231
d 22–28	0.70	0.63	0.018	0.015
Phase 1 (d 0–14)	0.63	0.75	0.062	0.004
Phase 2 (d 15–28)	0.70	0.64	0.033	0.037
Overall (0-28)	0.75	0.70	0.024	0.200

BW, body weight; IDP, indigestible dietary protein; SEM, standard error of the mean

1 Experimental diets with low IDP (LIDP) and high IDP (HIDP). Values are least square means; *n* = 40 pigs/treatment.

### Fecal consistency score

**Figure 1.**
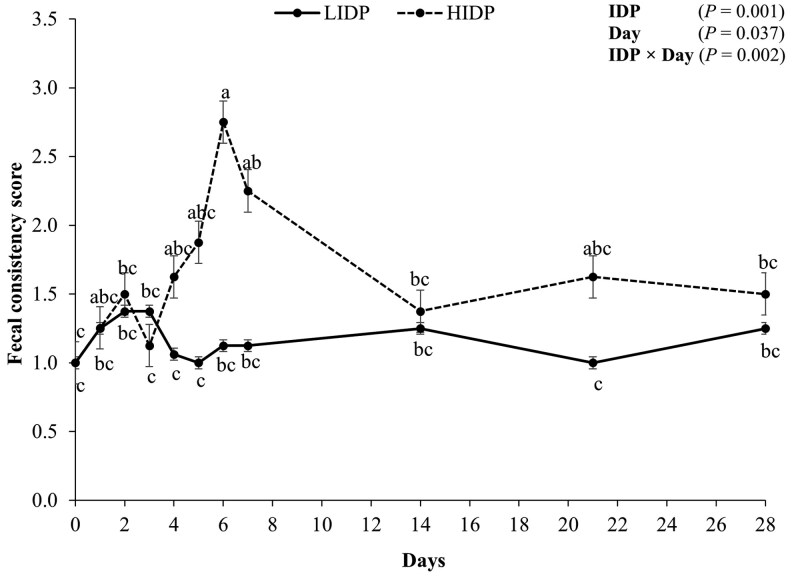
Fecal consistency score of piglets fed low (LIDP) or high (HIDP) indigestible dietary protein content. At eleven-time points (d 0, 1, 2, 3, 4, 5, 6, 7, 14, 21, and 28), fecal samples were collected per pen and visually scored. A score of 1 was assigned to normal (solid/firm) feces, a score of 2 to pasty feces, a score of 3 to moderately, loose fluid feces (moderate diarrhea), and a score of 4 to highly watery feces (severe diarrhea). Within days, treatments with different superscripts differ (*P *< 0.05). Values are least square means with error bars which represent the standard error of mean; *n* = 40 pigs/treatment.

Fecal consistency score of pigs fed LIDP or HIDP is presented in Figure 1. There were effects of IDP, day, and their interaction on FCS (*P *< 0.05), where FCS was higher in pigs fed HIDP and reached its peak score on d 6 compared to LIDP-fed pigs.

### Blood parameters

Blood parameters of pigs fed LIDP or HIDP are shown in Table 4. There were no IDP, Day, or interaction effects on ALB, IL-6, IL-1β, and ALP (*P *> 0.05). There was IDP × day effect on plasma haptoglobin, which tended to increase and decrease in LIDP and HIDP pigs, respectively, with experimental duration (*P *< 0.10). Plasma SOD was higher in pigs fed LIDP compared to HIDP (*P *< 0.05). There was an IDP × day interaction effect on plasma GSSG (*P *< 0.05), which was observed to increase and decrease in LIDP and HIDP pigs, respectively, with experimental duration. In addition, GSH:GSSG was observed to increase (*P *< 0.05), while GSH and GSSH tended to reduce (*P *< 0.10) over time.

**Table 4.** Blood parameters of piglets fed low or high indigestible dietary protein

	Diets				*P*-value		
Item	LIDP	HIDP	Day	SEM	IDP	Day	IDP × Day
**Serum albumin, g/L**							
d 9	34.9	35.4	35.1	1.314	0.288	0.736	0.458
d 28	33.2	36.0	34.6				
Mean	34.0	35.7					
**Serum interleukin-6, ρg/mL**							
d 9	31.5	41.3	36.4	4.013	0.601	0.314	0.240
d 28	32.5	28.7	30.6				
Mean	32.0	35.0					
**Serum interleukin-1β, ρg/mL**							
d 9	14.71	12.46	13.59	2.367	0.569	0.618	0.701
d 28	12.63	12.19	12.41				
Mean	13.67	12.33					
**Plasma haptoglobin, ng/mL**							
d 9	24.4	26.1	25.3	0.065	0.153	0.497	0.089
d 28	30.3	12.6	21.4				
Mean	27.3	19.3					
**Plasma alkaline phosphatase, IU/L**							
d 9	108	115	114	3.197	0.783	0.459	0.217
d 28	117	113	112				
Mean	113	114					
**Plasma superoxide dismutase, U/mol**							
d 9	0.16	0.13	0.15	0.011	0.032	0.742	0.709
d 28	0.16	0.12	0.14				
Mean	0.16^a^	0.13^b^					
**Reduced glutathione, µM**							
d 9	5.69	5.64	5.67	0.209	0.893	0.085	0.766
d 28	5.07	5.20	5.14				
Mean	5.38	5.42					
**Oxidized glutathione, µM**							
d 9	4.17^a^	4.24^ab^	4.21	0.446	0.110	0.059	0.048
d 28	4.82^a^	4.22^ab^	4.52				
Mean	4.50	4.23					
**GSH:GSSG**							
d 9	1.38	1.33	1.36	0.119	0.926	0.036	0.405
d 28	1.19	1.25	1.22				
Mean	1.29	1.29					

IDP, indigestible dietary protein; SEM, standard error of the mean

1 Experimental diets with low IDP (LIDP) and high IDP. Values are least square means; *n* = 8 pigs/treatment.

a, b In a column, means assigned different lowercase superscript letters are significantly different (*P *< 0.05).

### Principal component analysis of redox biomarkers and acute-phase protein in blood, fecal MPO, and growth performance measurements (d 9 and 28)

**Figure 2.**
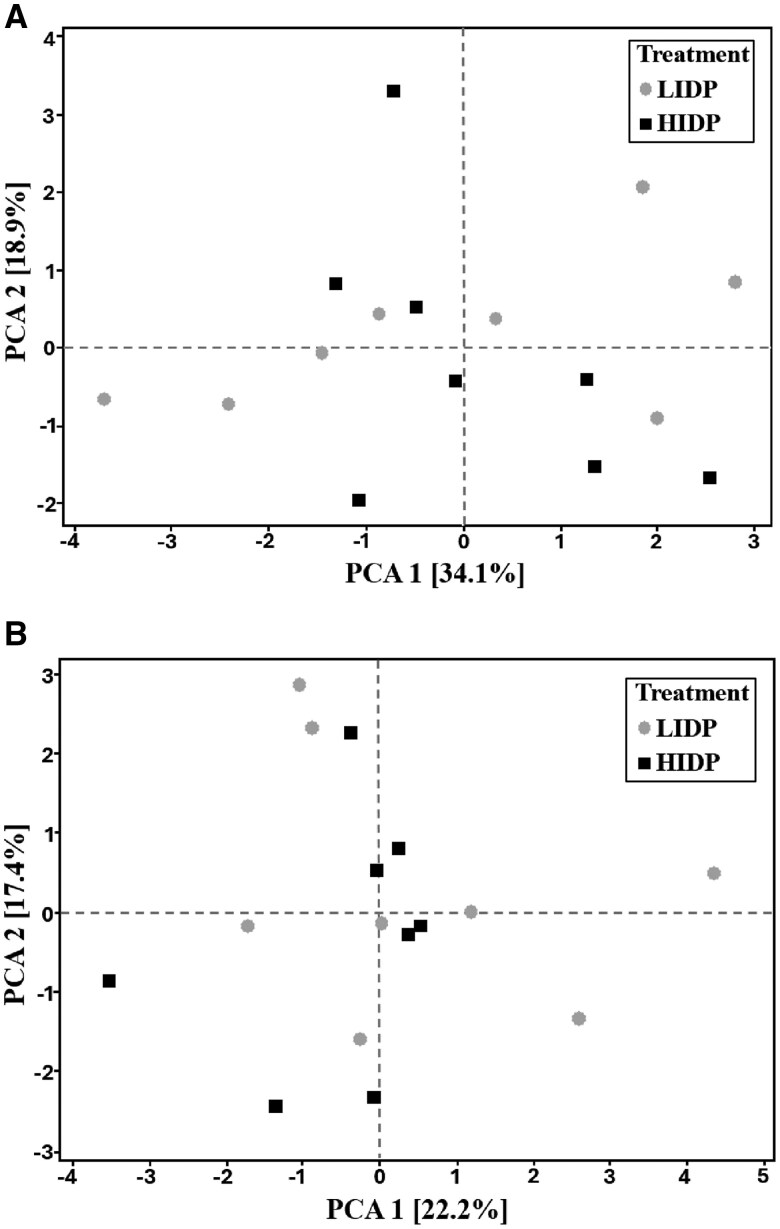
Principal component analysis score plot of plasma superoxide dismutase (SOD), plasma reduced glutathione (GSH), plasma oxidized glutathione (GSSG), plasma haptoglobin, plasma alkaline phosphatase (ALP), serum albumin (ALB), serum interleukin-1beta (IL-1β), and serum interleukin-6 (IL-6) on d9 (A), and the addition of average daily feed intake (ADFI), average daily gain (ADG), and gain: feed (G:F) responses on d28 (B). *n* = 8 pigs/treatment.

Principal components analysis, eigenvectors’ correlation matrix of factors to redox biomarkers and acute-phase protein in blood, fecal MPO (d9), and the addition of growth performance (d 28) measurements are presented in Tables 5 and 6, respectively, and their corresponding scree plot is presented in Figure 2A and B. On d 9, PC 1, 2, 3, and 4 accounted for 34.1, 18.9, 13.6, and 10% of total variance, respectively. However, the variation did not discriminate against any piglets fed either LIDP or HIDP. From the eigenvectors, PC1 had a relatively high correlation to plasma GSSG (*r* = 0.47), plasma GSH:GSSG (*r* = −0.47), and serum ALB (*r* = 0.41), PC2 had a relatively high correlation to plasma SOD (*r* = 0.50), PC3 had relative high correlation to serum IL-1β (*r *= 0.56), while PC4 had high correlation with serum IL-6 (*r* = 0.53) and fecal MPO (*r* = −0.55).

**Table 5.** Principal components, eigenvalues, percentage of variance, and cumulative percentages of variance described by components of redox biomarkers and acute-phase protein in blood and fecal myeloperoxidase (day 9), and the addition of growth performance (d 28)

Principal component	d 9			d 28		
	Eigenvalue	Proportion	Cumulative	Eigenvalue	Proportion	Cumulative
PC1	3.405	34.10	34.10	3.112	22.20	22.20
PC2	1.898	19.00	53.00	2.433	17.40	39.60
PC3	1.353	13.50	66.60	1.913	13.70	53.30
PC4	1.006	10.10	76.60	1.827	13.00	66.30
PC5	0.827	8.30	84.90	1.421	10.10	76.50
PC6	0.748	7.50	92.40	1.179	8.40	84.90
PC7	0.285	2.80	95.20	0.771	5.50	90.40
PC8	0.265	2.60	97.90	0.611,	4.40	94.80
PC9	0.211	2.10	100.00	0.485	3.50	98.20
PC10	0.001	0.00	100.00	0.139	1.00	99.20
PC11	–	–	–	0.071	0.50	99.70
PC12	–	–	–	0.036	0.30	100.00
PC13	–	–	–	0.003	0.00	100.00
PC14	–	–	–	0.000	0.00	100.00

PC, principal component; - indicates PC that were not analyzed in the model depending on the number of variables included in the PCA.

**Table 6.** Component matrix of redox biomarkers and acute-phase protein in blood, and fecal MPO (d 9) and the addition of growth performance (d 28)

Item	d 9					d 28						
	PC1	PC2	PC3	PC4	Communality	PC1	PC2	PC3	PC4	PC5	PC6	Communality
Plasma SOD, U/mL	−0.131	0.501	0.199	−0.204	0.63	0.253	−0.018	0.422	−0.177	−0.242	−0.382	0.85
Plasma GSH, µM	0.221	−0.484	−0.307	−0.298	0.83	0.175	−0.318	0.247	−0.098	0.515	0.198	0.90
Plasma GSSG, µM	0.473	−0.259	−0.002	−0.143	0.91	0.309	−0.275	−0.342	−0.232	0.178	−0.079	0.86
Plasma GSH: GSSG	−0.427	−0.223	−0.314	−0.182	0.88	−0.131	0.016	0.569	0.182	0.298	0.215	0.91
Plasma haptoglobin, mg/mL	0.291	0.307	−0.102	−0.254	0.55	−0.073	0.277	−0.304	−0.419	0.338	0.085	0.87
Plasma ALP, IU/L	0.348	0.323	−0.212	0.35	0.79	−0.016	−0.436	0.246	−0.171	−0.007	−0.419	0.84
Serum albumin g/L	0.413	0.119	0.362	−0.213	0.83	−0.394	0.028	0.153	0.179	−0.200	0.323	0.77
Serum IL-1β, ρg/mL	0.172	−0.405	0.555	0.095	0.84	0.152	−0.158	−0.07	−0.155	−0.594	0.267	0.77
Serum IL-6, ρg/mL	0.294	−0.025	−0.408	0.526	0.80	−0.014	0.487	−0.024	0.162	0.037	−0.37	0.79
Fecal MPO, nmol	0.187	0.143	−0.327	−0.546	0.60	−0.08	0.356	0.190	−0.203	0.037	−0.33	0.60
ADG_d28, kg	−	−	−	−	−	0.476	0.222	0.047	0.182	0.077	0.15	0.93
ADFI_d28, kg	−	−	−	−	−	0.145	0.307	0.221	−0.497	−0.024	0.333	0.97
G: F_d28, kg/kg	−	−	−	−	−	0.367	0.031	−0.125	0.513	0.107	−0.053	0.95
Final BW, kg	−	−	−	−	−	0.468	0.148	0.189	0.003	−0.167	0.147	0.87

BW, body weight; ADG, average daily gain; ADFI, average daily feed intake; ALP, alkaline phosphatase; G:F, gain:feed; GSH, reduced glutathione; GSSG, oxidized glutathione; *IL*, interleukin; MPO, myeloperoxidase; SOD, superoxide dismutase; −, data not collected on d 9 and not included in the component matrix.

*n* = 8 pigs/treatment

On d 28, PC 1, 2, 3, 4, 5, and 6 accounted for 22.2, 17.4, 13.7, 13.0, 10.1, and 8.4% of total variance, respectively. However, the variation did not discriminate against any piglets fed either LIDP or HIDP. From the eigenvectors, PC1 had a relatively high correlation to ADG (*r* = 0.48), and final BW (*r* = 0.47), PC2 had a relatively high correlation to plasma ALP (*r* = −0.44) and serum IL-6 (*r* = 0.49), PC3 had a relatively high correlation to plasma GSH:GSSG (*r* = 0.57), PC4 had high correlation with G:F (*r* = 0.51), PC5 had a relatively high correlation to plasma GSH (*r* = 0.52) and serum IL-1β (*r* = −0.59), and PC6 had a relatively high correlation to plasma ALP (*r* = −0.42).

### Hindgut ammonia–nitrogen concentrations

**Figure 3.**
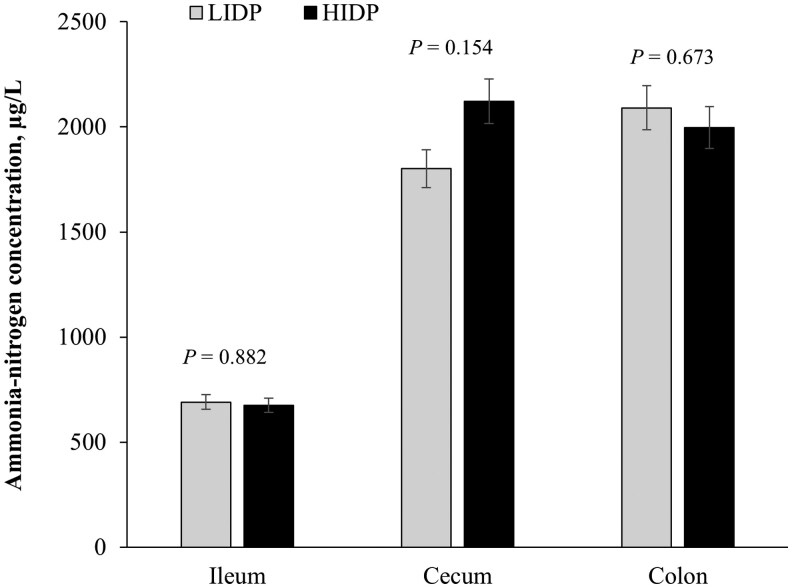
Ammonia-nitrogen concentrations in ileal, cecal, and colonic digesta collected on d 9 from piglets fed low (LIDP) or high indigestible dietary protein (HIDP). Values are least square means with error bars which represent the standard error of mean; *n* = 8 pigs/treatment. Probability values corresponding to main effects of IDP on was not significant (*P *> 0.10).

Ammonia–nitrogen concentrations in ileal, cecal, and colonic digesta of piglets fed LIDP and HIDP are presented in Figure 3. There was no effect of IDP on ileal, cecal, and colonic NH_3_-N concentrations (*P *> 0.05).

### Volatile fatty acid profile and other fermentation metabolites in distal gut digesta

The volatile fatty acid profile and other protein fermentation metabolites in the ileal, cecal, and colonic digesta of piglets fed LIDP and HIDP are shown in Table 7. There was no effect of IDP on the concentrations of acetic acid, propionic acid, and butyric acid in ileal, cecal, and colonic digesta (*P *> 0.05). Compared to HIDP, LIDP reduced valeric acid concentration in the colonic digesta (*P *< 0.05) but tended to decrease it in the cecal digesta (*P *< 0.10). Additionally, LIDP tended to decrease the concentration of isovaleric acid in the ileal, cecal, and colonic digesta (*P *< 0.10), and only showed a tendency to decrease isobutyric acid in the cecal digesta compared to LIDP (*P *< 0.10). In the ileal digesta, LIDP decreased the concentrations of tryptamine and choline (*P *< 0.05), increased cystathionine and sarcosine (*P *< 0.05), and tended to increase spermine (*P *< 0.10). In the cecal digesta, LIDP increased the concentrations of cadaverine, spermine, allantoin, creatinine, and N1-acetylspermidine (*P *< 0.05), reduced phenylethylamine, tryptamine and choline (*P *< 0.05), and tended to increase and decrease *N-*acetylputrescine and tryptamine, respectively (*P *< 0.10). In the colonic digesta, the concentration of choline was reduced in LIDP-fed pigs compared to those fed HIDP (*P *< 0.05).

**Table 7.** Volatile fatty acids profile and other protein fermentation metabolites concentrations in fresh mass of ileal, cecal, and colonic digesta collected on day 9 from pigs fed LIDP and HIDP diets

	Ileal digesta				Cecal digesta				Colonic digesta			
Item	LIDP	HIDP	SEM	*P*-value	LIDP	HIDP	SEM	*P*-value	LIDP	HIDP	SEM	*P*-value
**Short-chain fatty acids, mmol/L**												
Acetic acid	91.3	99.7	0.018	0.502	50.8	47.1	0.001	0.263	54.2	51.4	0.001	0.821
Propionic acid	32.0	32.0	<0.001	0.954	18.0	17.7	0.001	0.796	24.9	22.1	0.001	0.304
Butyric acid	44.2	18.1	0.053	0.110	6.36	6.42	0.001	0.942	8.80	8.86	0.001	0.959
**Branched-chain fatty acids, mmol/L**												
Isobutyric acid	4.92	6.08	0.002	0.107	1.34	2.53	0.001	0.081	1.95	2.49	0.001	0.203
Valeric acid	3.43	3.89	<0.001	0.503	1.79	3.07	0.001	0.066	0.99	1.14	0.001	0.018
Isovaleric acid	0.65	0.81	0.001	0.088	0.93	1.40	0.001	0.076	0.84	1.19	0.001	0.083
**Biogenic amines, µmol/L**												
Agmatine	0.07	0.14	0.01	0.197	0.60	0.41	0.12	0.142	0.20	0.17	0.13	0.695
Dopamine	–	–	–	–	5.77	3.33	1.33	0.202	0.16	0.22	0.00	0.750
Ethanolamine	98.3	126	1.34	0.561	240	267	1.13	0.539	200	186	1.37	0.873
Histamine	0.66	1.85	0.01	0.148	13.6	31.3	1.90	0.377	13.8	25.0	2.08	0.527
Methylamine	–	–	–	–	360	277	1.18	0.275	37.5	51.4	1.84	0.718
*N*-Acetylputrescine	2.42	5.13	1.45	0.175	54.0	27.9	1.28	0.071	12.8	11.1	1.47	0.804
Phenylethylamine	–	–	–	–	0.16	0.46	0.00	0.003	0.80	1.08	0.00	0.634
Cadaverine	48.1	36.2	1.77	0.715	839	513	1.10	0.002	507	286	9.27	0.213
Putrescine	159	241	1.44	0.436	519	390	1.15	0.171	123	155	1.31	0.545
Serotonin	0.07	0.09	0.02	0.571	0.42	0.32	0.04	0.130	0.63	0.62	0.00	0.972
Spermidine	62.0	39.4	1.33	0.278	294	232	1.11	0.117	131	92.5	1.51	0.560
Spermine	4.41	2.11	0.87	0.082	6.96	2.77	1.16	0.001	0.36	0.80	0.00	0.357
Tryptamine	0.03	0.08	0.01	0.048	7.12	31.1	1.44	0.013	78.7	82.9	1.19	0.835
Tyramine	–	–	–	–	46.5	139	1.49	0.072	62.0	137	1.40	0.121
**Non-biogenic amines, µmol/L**												
Choline	175	570	1.31	0.001	95.2	188	1.08	<0.001	64.2	118	1.26	0.079
Cystathionine	1.86	0.64	0.49	0.021	1.07	1.36	0.22	0.373	0.87	2.07	0.49	0.104
Allantoin	91.7	68.1	1.18	0.236	56.4	24.3	1.21	0.002	15.5	26.6	1.61	0.433
Creatine	190	113	38.0	0.173	129	13.6	1.66	0.008	3.42	4.18	1.31	0.646
*N*1-Acetylspermidine	11.6	14.9	1.49	0.642	124	85.8	1.12	0.040	51.1	30.9	1.39	0.295
Sarcosine	0.66	0.38	0.13	0.045	3.40	2.52	0.64	0.349	2.62	2.30	0.00	0.704
Taurine	1.83	2.03	0.16	0.398	22.8	21.5	1.09	0.612	20.4	21.7	0.16	0.706

HIDP, high indigestible dietary protein; LIDP, low indigestible dietary protein; SEM, standard error of means.

Values are least square means; *n* = 8 pigs/treatment.

### Fecal myeloperoxidase activity

**Figure 4.**
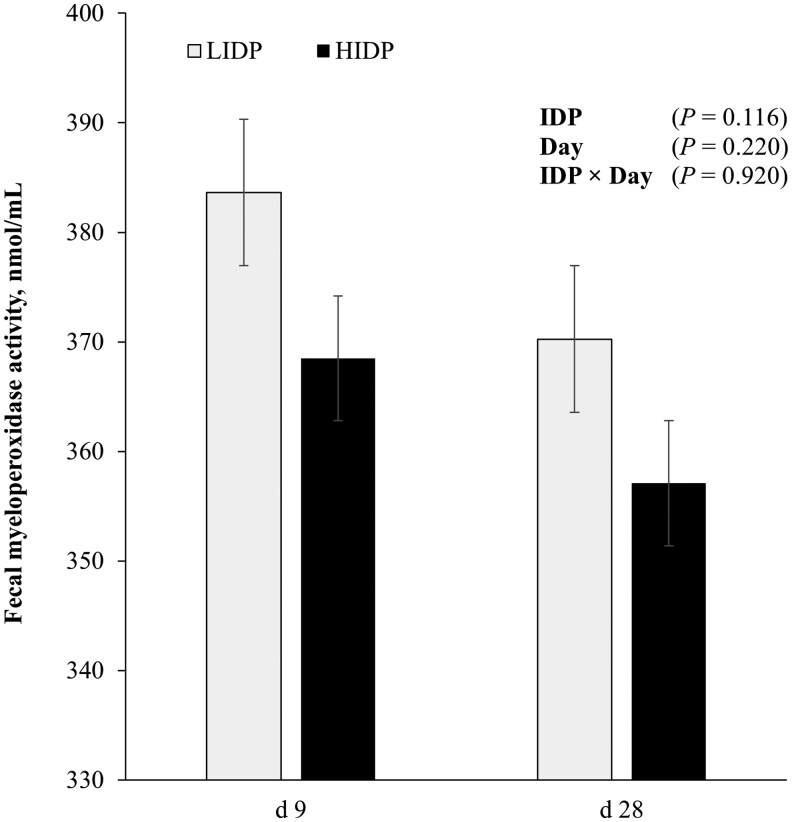
Fecal myeloperoxidase activity (MPO) on d 9 and 28 in piglets fed a low (LIDP) or high indigestible dietary protein (HIDP) content. The fecal MPO values refers to fresh mass. Values are least square means with error bars which represent the standard error of mean; *n* = 40 pigs/treatment.

Fecal MPO of pigs fed LIDP and HIDP is presented in Figure 4. There were no effects of IDP or Day on the activity of fecal MPO (*P *> 0.05).

## Discussion

The objective of the present study was to examine the effect of low or high IDP on the growth performance, immune status, inflammatory status, hindgut fermentation metabolites, and diarrhea severity in weaning pigs. The presence of undigested DP in the GIT is speculated to have negative effects on the GIT in nursery pigs (Andersen, 2018), which in turn affect their growth performance. Herein, the amount of DP that is undigested in the foregut but becomes available for microbial fermentation in the hindgut is considered IDP. However, IDP is an emerging dietary concept, and information on its in vivo effect on growth performance and other gut health indicators in piglets is lacking. To our knowledge, this is the first study to examine the effect of IDP using plant-based protein sources in newly weaned pigs. It is important to note that in this study, IDP was calculated as the difference between total CP and SID CP. As published SID values are largely obtained from growing/finishing pigs, the values for IDP are likely underestimated, especially for the HIDP diet which contains DDGS and canola meal.

### Effect of IDP content on growth performance of weaning pigs

In the present study, diets were formulated to contain low or high IDP content while maintaining similar total protein (2.7% and 4.2% IDP). Because diets were similar in nutrient content, except for IDP, they would not be expected to impact the growth performance of piglets. Moreover, IDP content of diets was varied by altering the inclusion of only plant-based protein sources. Therefore, all observed effects can be assumed to be an effect of the IDP content without the confounding effect of animal vs. plant-based protein sources, which differs from previous studies examining protein content in weaned pigs (Wang et al., 2022; Rodrigues et al., 2022; de Oliveira et al., 2025). Overall, pigs fed HIDP diet were observed to have lower ADG compared to those fed LIDP; however, neither diet affected overall ADFI, G:F, or final BW. In agreement with the present findings, our recent meta-analytical report showed that IDP index negatively influenced ADG of pigs, given the association with hindgut fermentation of protein (de Oliveira et al., 2025). Similarly, overall BW, BW gain, and feed: gain were reportedly improved in broiler chickens fed LIDP compared to HIDP, which was formulated by partially replacing SBM with corn DDGS and corn gluten meal (Bryan et al., 2019). In contrast to our findings, Piel et al. (2007) reported that feeding a high IDP diet of 4% using peas as a partial replacement for fish protein and cereals did not affect ADFI or slaughter BW of weaning pigs compared to those fed diets containing a low IDP of 2.1%. The deviation of the present report from the findings by Piel et al. (2007) is likely due to the mixture of animal- and plant-based protein ingredients with increasing dietary fiber in that study. At a similar DP level of 22%, Bekebrede et al. (2022) reported that the addition of a less digestible DP source, collagen, reduced weight gain of pigs compared to those fed a more digestible protein source, whey. This implies that feeding diets containing LIDP sources could improve the growth performance of weaning pigs. In the current study, the reduced overall ADG in HIDP-fed pigs partly confirms our hypothesis that HIDP would reduce the growth performance of nursery pigs.

### Effect of IDP content on fecal consistency score of weaning pigs

Products of proteolytic fermentation have been shown to induce intestinal inflammation by activating NF-κB signaling and promotion of chloride ion secretion and PWD in piglets (Kröger et al., 2013; Yin et al., 2021). In the present study, we observed a higher FCS in pigs fed HIDP compared to those fed LIDP, on d 6 post-weaning. Fecal consistency score has been used as an indicator of diarrhea severity in several studies (Liu et al., 2010; Wen et al., 2018; Eriksen et al., 2024). Depending on the scoring system used, an FCS greater than the score for soft or pasty feces is usually considered severe diarrhea (Renaud et al., 2020; Eriksen et al., 2024; Erinle et al., 2025). Numerous studies involving high protein diets, usually >20% CP, demonstrated their capacity to induce diarrhea (Yin et al., 2021; Xia et al., 2022; Kim et al., 2023). Surprisingly, other studies reported that high protein diets ranging from 20% to 25% CP were not a predisposing factor for diarrhea severity compared to low protein diets (Rodrigues et al., 2021; Correia et al., 2023; Diether et al., 2024; Pearce et al., 2024). The inconsistent effects of DP on diarrhea severity suggest it might not be relevant dietary factor causing diarrhea. Rather than the DP levels, the use of IDP may be a better indicator as less digestible protein diets present a considerable amount of undigested protein to the hindgut, where it becomes a precursor for nutritional diarrhea in weaning pigs. Of note, IDP was demonstrated to increase PWD in piglets (Gilbert et al., 2022). Thus, the higher FCS observed in pigs fed HIDP in the present study supports our hypothesis that feeding a HIDP would promote diarrhea severity in newly weaned pigs.

### Effect of IDP content on pro-inflammatory cytokines, acute-phase protein, oxidative status, and intestinal inflammation in weaning pigs

The immature and developing GIT of weaning pigs experiences a transient local inflammatory response during early post-weaning periods characterized by the elevation of pro-inflammatory cytokines (McCracken et al., 1995; McCracken et al., 1999; de Groot et al., 2021) and haptoglobin (McCracken et al., 1999). In addition to weaning, dietary factors, including DP content (Limbach et al., 2021), dietary fiber supplementation (Che et al., 2012a, 2012b), or undigested DP (Long et al., 2021) plays critical roles in modulating inflammatory responses. For example, feeding high levels of IDP content is speculated to upregulate pro-inflammatory cytokines, which could decrease gut integrity in nursery pigs (Long et al., 2021). In the present study, we did not observe any adverse effect of HIDP diet on the alteration of pro-inflammatory cytokines, namely IL-6 and IL-1β. The absence of IDP effect on the measured pro-inflammatory cytokines may be reflect limitations in timing of the relevant sample collected which did not coincide with the period when FCS was most impact. Given the preceding, a similar effect would be expected on the oxidative status of the piglets. However, the mechanism through which IDP influences oxidative status in piglets is less clear. Increasing DP from 21% to 24% is thought to promote proteolytic fermentation and may cause increased inflammation (Diether and Willing, 2019), potentially provoking oxidative stress. In other instances, increasing DP levels from 11% to 15% CP (Liu et al., 2025) or from 14% to 22% CP (Liu et al., 2023) has been demonstrated to improve enzymatic antioxidant defense mechanism by upregulating serum glutathione peroxidase, plasma catalase, GSH, and SOD in pigs. In the present study, prolonged feeding of LIDP increased plasma GSSG and tended to increase plasma haptoglobin compared to HIDP. While a shift towards upregulated plasma GSSG suggests an imbalanced antioxidant status, pigs fed LIDP diet did not suffer oxidative stress, as evidenced by similar plasma GSH and GSH:GSSG levels compared to those fed HIDP diet. In fact, LIDP-fed had upregulated plasma SOD compared to the HIDP-pigs. Amongst others, superoxide dismutase is dubbed the most potent endogenous antioxidant enzyme responsible for the dismutation of reactive oxygen (Ighodaro and Akinloye, 2018). Interestingly, both plasma GSSG and SOD showed a relatively high correlation with the first two PC, which mainly reflect oxidative status and, therefore, could summarized the impact of IDP in weaning pigs. This was further supported by the absence of IDP effect on blood inflammatory cytokines and acute-phase protein in the present study.

The negative impact of undigested DP and its metabolites, including ammonia, phenols, and resistant protein, has been linked to increased thinning of colonic mucus layer in rats and humans (Lin and Visek, 1991; Santoro et al., 1999; McCall et al., 2009). Notably, Piel et al. (2007) fed a diet containing approximately 30% crude protein (CP) and high IDP concentration of 4%, which resulted in an increased erosion of the ileal mucus layer in weanlings, thereby exposing them to colonization susceptibility or inflammation. In the current study, there was no IDP effect on gut inflammation, as evidenced by the similar fecal MPO concentrations among pigs fed either LIDP or HIDP. Fecal MPO is a lysosomal component of polymorphonuclear leukocytes and is associated with intestinal inflammation (Pearce et al., 2013). The reason for the lack of IDP effect on intestinal inflammation is quite understandable as this follows with other inflammatory biomarkers which is not impacted by the IDP levels in present study.

### Effect of IDP content on short-chain fatty acids, branched-chain fatty acids, biogenic amines and precursors, and ammonia–nitrogen concentrations in weaning pigs

To examine the fate of varying IDP levels reaching the hindgut and their relevance on FCS, oxidative status, and inflammatory biomarkers, we further determined the concentration of hindgut fermentation metabolites in the ileal, cecal, and colonic digesta. Undigested DP reaching the hindgut is fermented to produce short-chain fatty acids (SCFA) and branched-chain fatty acids (BCFA), particularly following a depletion in the carbohydrate content in the digesta (Windey et al., 2012; Diether and Willing, 2019). Gastric infusion of SCFA have been reported to increase the abundance of tight junction protein and beneficial bacteria in the upper GIT while reducing IL-1β in the colon of weaned piglets (Diao et al., 2019). On the other hand, BCFA are particularly produced from the fermentation of branched-chain amino acids and have been sought as reliable bio-indicators of proteolytic fermentation (Diether and Willing, 2019). In the present study, there was no IDP effect on the concentration of SCFA, including acetate, propionate, and butyrate in ileal, cecal, and colonic digesta. However, HIDP increased valerate in the colonic digesta, and tended to increase the concentrations of short BCFA, including isovalerate throughout the distal GIT, isobutyrate and valerate in the cecal digesta compared to the LIDP. Unfortunately, the evidence of BCFA effects in pigs is very scarce in literature and mostly conflicting in many animal species. The beneficial effects of BCFA on gut health have been reported (Kennedy et al., 2021; Fang et al., 2025); however, these have been mostly associated with long-chain BCFA (Gozdzik et al., 2023; Zhang et al., 2025). Short BCFA has been implicated in the perturbation of gut microbiota, depression, anorexia nervosa, obesity, reduced viability of colonocytes, and inflammatory bowel disease (Newgard et al., 2009; Szczesniak et al., 2016; Borghi et al., 2017; Lee et al., 2021; Yang et al., 2025). The tendency of HIDP to increase BCFA in the present study suggests its possibility to exacerbate hindgut health of pigs and thereby worsen weaning outcomes.

Indigestible DP, peptides, or amino acids in the hindgut also undergo decarboxylation reactions to produce biogenic amines. This process is chiefly modulated by proteolytic bacteria in the distal GIT. Depending on the dose, biogenic amines can be either beneficial or detrimental to hindgut health (Silva et al., 2020). However, information on the dose-dependent toxicity of biogenic amines in the pigs is scant. In any case, IDP appeared to have dynamic effects in the distal GIT, with more protein fermentation metabolites being influenced in the cecal region. In the present study, HIDP increased the concentration of tryptamine in both ileal and cecal digesta, as well as phenylethylamine in the cecal digesta. Tryptamine and phenylethylamine have been demonstrated in vitro to be involved in necrotic cell death via their cytotoxic mode of action (Del Rio et al., 2020). Furthermore, feeding LIDP tended to increase the concentration of spermine in ileal digesta, while significantly increasing cadaverine and spermine in the cecal digesta. Meanwhile, HIDP-fed pigs had higher *N*-acetylputrescine and tended to have higher tyramine concentrations in their cecal digesta. Oral supplementation of spermine in suckling pigs was reported to improve gut histomorphometry at weaning (van Wettere et al., 2016). In mice, cadaverine has been reported to possess tumor-suppressive capabilities (Kovács et al., 2019). Contrastingly, the cytotoxicity of spermine, cadaverine, and tyramine towards intestinal cell culture has also been reported (Del Rio et al., 2017, 2018, 2019). While we are aware that biogenic amines have dose-dependent effects, we could not establish whether the concentrations of biogenic amines we found in the present study are at the level that might augment or provoke hindgut health.

In the present study, LIDP-fed pigs were observed to have increased concentrations of non-biogenic amines, including allantoin, creatine, and N1-acetylspermidine, in the cecal digesta. Additionally, cystathionine and sarcosine were found to be higher in the ileal digesta. In pigs, allantoin is synthesized from uric acid—a waste product of purine metabolism. In mice induced with gastric ulcers, allantoin was shown to possess anti-inflammatory, antioxidant, and cytoprotective properties (da Silva et al., 2018). In addition to the positive effects of allantoin, creatine has been reported to be a major metabolite required for amino acid metabolism and methyl balance in neonatal piglets (Brosnan et al., 2009). Cystathionine is an essential intermediate in the conversion of homocysteine produced from methionine to cysteine via the irreversible transsulfuration pathway. In addition to its antioxidant effect, cysteine was shown to play a critical modulatory role in suppressing pro-inflammatory responses in the gut mucosa (Xu et al., 2014; Kim et al., 2009; Jiao et al., 2022). On the other hand, HIDP-fed pigs had higher choline concentrations in the ileal and cecal digesta and tended to be higher in the colonic digesta. Supplementation of choline to weaning pigs has been demonstrated to modulate growth performance and intestinal inflammation (Qiu et al., 2021). However, this did not translate to improved overall growth performance in the HIDP fed pigs. Canola meal has been shown to contain higher concentration of sinapine which could be hydrolyzed to choline in the small intestine and subsequently to trimethylamine/trimethylamine N-oxide in hindgut microbes (Chen et al., 2019). However, total choline in canola meal has been reported to be unavailable compared to other plant-based protein sources like soybean and peanut meals (Emmert and Baker, 1997). Hence, the higher choline concentration in the hindgut could be due to the inclusion of canola in the HIDP which is not present in the LIDP.

Ammonia nitrogen, another product of fermentation of protein by resident gut microbes, is often positively correlated with DP level and fecal score (Opapeju et al., 2008; Zhao et al., 2021). In the present study, there was no IDP effect on NH_3_-N concentrations in the ileal, cecal, and colonic digesta. In disagreement with our findings. Sung et al. (2023) reported an increased concentration of ileal IDP and higher fecal nitrogen concentrations in growing pigs fed a high IDP diet. While the absence of IDP effect on NH_3_–N concentrations in the distal GIT in the present study was unexpected, the probable reasons could be due to the healthy status of the piglets or the lack of immune stimulation in the pigs. Taken together, the varying IDP levels did not cause an alteration in intestinal permeability, given the absence of effect on the serum pro-inflammatory ­cytokines and fecal MPO activity; however, an HIDP level showed a capacity to impact antioxidant status and some potentially harmful protein fermentation metabolites in healthy nursery pigs.

## Conclusions

Overall, HIDP, containing 4.2% IDP content, increased FCS and reduced overall ADG, while overall ADFI, G:F, and final BW remained unaffected. In addition, HIDP reduced plasma antioxidant enzyme by reducing SOD concentrations, while prolong feeding of LIDP increased plasma GSSG without altering plasma GSH:GSSG. Furthermore, feeding HIDP tended to increase the concentrations of short BCFA in the distal GIT without affecting serum inflammatory biomarkers and NH_3_-N concentrations. These results suggest that IDP should be considered when developing diets for improved performance, reduction of diarrhea severity, and antioxidative resilience in nursery pigs.

## Data Availability

Data is available upon reasonable request to the corresponding author.

## Abbreviations:

AA: amino acid
ADFI: average daily feed intake
ADG: average daily gain
ALB: albumin
ALP: alkaline phosphatase
BCFA: branched-chain fatty acids
BW: body weight
CP: crude protein
DP: dietary protein
DM: dry matter
FCS: fecal consistency score
G:F: gain:feed
GIT: gastrointestinal tract
GSH: reduced glutathione
GSSG: oxidized glutathione
HIDP: high indigestible dietary protein
IDP: indigestible dietary protein
IL: interleukin
LIDP: low indigestible dietary protein
ME: metabolizable energy
MPO: myeloperoxidase activity
NE: net energy
NH_3_-N: ammonia-nitrogen
PC: principal components
PUN: plasma urea nitrogen
PWD: post-weaning diarrhea
SID: standardized ileal digestible
SOD: superoxide dismutase
VFA: volatile fatty acids

## References

[skaf372-B1] Andersen L. S. 2018. Cut indigestible protein—and do piglet health a favour. https://www.pigprogress.net/pigs/cut-indigestible-protein-and-do-piglet-health-a-favour/

[skaf372-B2] Bekebrede A. F. , Noorman L., Keijer J., de Boer V. C., Gerrits W. J. 2022. Functional metabolic capacity of pig colonocytes is differentially modulated by fermentable fibre and poorly digestible protein. Animal. 16(11):100625. 10.1016/j.animal.2022.100625 36265188

[skaf372-B3] Bloomer S. A. 2018. Combinational Use of Na-Butyrate and Phytobiotics on Growth Performance and Intestinal Health of Nursery Pigs. North Carolina State University. https://repository.lib.ncsu.edu/handle/1840.20/35080

[skaf372-B4] Borghi E. , Borgo F., Severgnini M., Savini M. N., Casiraghi M. C., Vignoli A. 2017. Rett syndrome: a focus on gut microbiota. Int. J. Mol. Sci. 18(2):344. 10.3390/ijms18020344 28178201 PMC5343879

[skaf372-B5] Brosnan J. T. , Wijekoon E. P., Warford-Woolgar L., Trottier N. L., Brosnan M. E., Brunton J. A., Bertolo R. F. 2009. Creatine synthesis is a major metabolic process in neonatal piglets and has important implications for amino acid metabolism and methyl balance. J. Nutr. 139(7):1292–-1297. 10.3945/jn.109.105411 19474158

[skaf372-B6] Bryan D. D. L. S. , Abbott D. A., Classen H. L. 2019. The influence of indigestible protein on the performance and meat quality of broilers vaccinated for coccidiosis. Poult. Sci. 98(10):4815–-4828. 10.3382/ps/pez216 30995312

[skaf372-B7] Canadian Council on Animal Care. 2009. The care and use of farm animals in research, teaching and testing. Ottawa: CCAC; p. 12–15.

[skaf372-B8] Che T. M. , Johnson R. W., Kelley K. W., Dawson K. A., Moran C. A., Pettigrew J. E. 2012a. Effects of mannan oligosaccharide on cytokine secretions by porcine alveolar macrophages and serum cytokine concentrations in nursery pigs. J. Anim. Sci. 90(2):657–668. 10.2527/jas2011-4310. 21984710

[skaf372-B9] Che T. M. , Song M., Liu Y., Johnson R. W., Kelley K. W., Van Alstine W. G., Dawson K. A., Pettigrew J. E. 2012b. Mannan oligosaccharide increases serum concentrations of antibodies and inflammatory mediators in weanling pigs experimentally infected with porcine reproductive and respiratory syndrome virus. J. Anim. Sci. 90(8):2784–2793. 10.2527/jas.2011-4518 22367071 PMC7110021

[skaf372-B10] Chen H. , Peng L., Pérez de Nanclares M., Trudeau M. P., Yao D., Cheng Z., Urriola P. E., Mydland L. T., Shurson G. C., Overland M. et al 2019. Identification of sinapine-derived choline from a rapeseed diet as a source of serum trimethylamine N-oxide in pigs. J. Agric. Food Chem. 67(27):7748–7754. 10.1021/acs.jafc.9b02950 31203621

[skaf372-B11] Correia A. M. , Genova J. L., Saraiva A., Rocha G. C. 2023. Effects of crude protein and non-essential amino acids on growth performance, blood profile, and intestinal health of weaned piglets. Front. Vet. Sci. 10:1243357. 10.3389/fvets.2023.1243357 38098993 PMC10720428

[skaf372-B12] da Silva D. M. , Martins J. L. R., de Oliveira D. R., Florentino I. F., da Silva D. P. B., Dos Santos F. C. A., Costa E. A. 2018. Effect of allantoin on experimentally induced gastric ulcers: Pathways of gastroprotection. Eur. J. Pharmacol. 821:68–78. 10.1016/j.ejphar.2017.12.052 29277718

[skaf372-B14] de Groot N. , Fariñas F., Cabrera-Gómez C. G., Pallares F. J., Ramis G. 2021. Weaning causes a prolonged but transient change in immune gene expression in the intestine of piglets. J. Anim. Sci. 99(4):skab065. 10.1093/jas/skab065 33640983 PMC8051849

[skaf372-B15] de Oliveira M. J. K. , Babatunde O. O., Rodrigues L. A., Erinle T. J., Htoo J. K., Mendoza S. M., Columbus D. A. 2025. Development of an indigestible dietary protein index to investigate the effects of dietary protein content in post-weaning pigs. J. Anim. Sci. 103:skae374. 10.1093/jas/skae374 PMC11705088 39657758

[skaf372-B16] Del Rio B. , Redruello B., Linares D. M., Ladero V., Fernandez M., Ruas-Madiedo P., Martin M. C., Alvarez M. A. 2017. The dietary biogenic amines tyramine and histamine show synergistic toxicity towards intestinal cells in culture. Food Chem. 218:249–255. 10.1016/j.foodchem.2016.09.046 27719906

[skaf372-B17] Del Rio B. , Redruello B., Linares D. M., Ladero V., Ruas-Madiedo P., Fernandez M., Martin M. C., Alvarez M. A. 2018. Spermine and spermidine are cytotoxic towards intestinal cell cultures, but are they a health hazard at concentrations found in foods? Food Chem. 269:321–326. 10.1016/j.foodchem.2018.06.148 30100441

[skaf372-B18] Del Rio B. , Redruello B., Linares D. M., Ladero V., Ruas-Madiedo P., Fernandez M., Martin M. C., Alvarez M. A. 2019. The biogenic amines putrescine and cadaverine show in vitro cytotoxicity at concentrations that can be found in foods. Sci. Rep. 9(1):120. 10.1038/s41598-018-36239-w 30644398 PMC6333923

[skaf372-B19] Del Rio B. , Redruello B., Fernandez M., Martin M. C., Ladero V., Alvarez M. A. 2020. The biogenic amine tryptamine, unlike β-phenylethylamine, shows in vitro cytotoxicity at concentrations that have been found in foods. Food Chem. 331:127303. 10.1016/j.foodchem.2020.127303 32562979

[skaf372-B20] Deng Z. , Duarte M. E., Kim S. W. 2023. Efficacy of soy protein concentrate replacing animal protein supplements in mucosa-associated microbiota, intestinal health, and growth performance of nursery pigs. Anim. Nutr. 14:235–248. 10.1016/j.aninu.2023.06.007 37600837 PMC10432921

[skaf372-B21] Diether N. E. , Kommadath A., Fouhse J. M., Zijlstra R. T., Stothard P., Willing B. P. 2024. Increased dietary protein rather than fiber supports key metabolic and intestinal tissue functions in pigs, without increasing post-weaning diarrhea. Am. J. Physiol. Gastrointest. Liver Physiol. 327(6):G818–G831. 10.1152/ajpgi.00146.2024 39406387

[skaf372-B22] Diether N. E. , Willing B. P. 2019. Microbial fermentation of dietary protein: an important factor in diet–microbe–host interaction. Microorganisms. 7(1):19. 10.3390/microorganisms7010019 30642098 PMC6352118

[skaf372-B23] Diao H. , Jiao A. R., Yu B., Mao X. B., Chen D. W. 2019. Gastric infusion of short-chain fatty acids can improve intestinal barrier function in weaned piglets. Genes Nutr. 14(4):4–16. 10.1186/s12263-019-0626-x 30761185 PMC6359775

[skaf372-B24] Emmert J. L. , Baker D. H. 1997. A chick bioassay approach for determining the bioavailable choline concentration in normal and overheated soybean meal, canola meal and peanut meal. J. Nutr. 127(5):745–752. 10.1093/jn/127.5.745 9164996

[skaf372-B25] Eriksen E. Ø. , Sejersen M. F., Pedersen K. S. 2024. The cotton swab method: an accurate and less invasive way to assess fecal consistency in weaned pigs. BMC Vet. Res. 20(1):47. 10.1186/s12917-024-03888-1 38310282 PMC10837864

[skaf372-B26] Erinle T. J. , Babatunde O. O., Htoo J. K., Mendoza S. M., Columbus D. A. 2025. Dietary fibre fractions supplementation modulates pro-inflammatory cytokines, hindgut fermentation metabolites, and fecal consistency score in nursery pigs. Can. J. Anim. Sci. 105:1–15. 10.1139/cjas-2024-0123

[skaf372-B27] Evonik. 2021. AMINODat^®^6.0Feed Raw Material Database. Hanau-Wolfgang, Germany: Evonik Nutrition & Care GmbH.

[skaf372-B28] Fang L. H. , Jin Y. H., Do S. H., Hong J. S., Kim B. O., Han T. H., Kim Y. Y. 2019. Effects of dietary energy and crude protein levels on growth performance, blood profiles, and nutrient digestibility in weaning pigs. Asian-Australas. J. Anim. Sci. 32(4):556–563. 10.5713/ajas18.0294 30145868 PMC6409451

[skaf372-B19682969] Fang X. , Wang Z., Chen Q., Du Y., Sun H., Liu H., Feng Ye., Li Z., Teng T., and Shi B. 2025. Protective effect of the branched short‐chain fatty acid isobutyrate on intestinal damage in weaned piglets through intestinal microbiota remodeling. J. Sci. Food Agric. 105(3):1556–1568. 10.1002/jsfa.13930 39412364

[skaf372-B29] Foroutan A. , Fitzsimmon C., Mandal R., Piri-Moghadam H., Zheng J., Guo A. C., Li C., Guan L. L., Wishart D. S. 2020. The bovine metabolone. Metabolites. 10(6):233. 10.3390/metabo10060233 32517015 PMC7345087

[skaf372-B30] Foroutan A. , Guo A. C., Vazquez-Fresno R., Lipfert M., Zhang L., Zheng J., Badran H., Budinski Z., Mandal R., Ametaj B. N. et al 2019. Chemical composition of commercial cow’s milk. J. Agric. Food Chem. 67(17):4897–4914. 30994344 10.1021/acs.jafc.9b00204

[skaf372-B33] Gaskins H. R. 2000. Intestinal bacteria and their influence on swine growth. In: Swine Nutrition, 2nd Edition. CRC Press, Boca Raton, FL, USA. 585-608.

[skaf372-B34] Gilbert M. S. , Noorman L., van der Hee B., Gerrits W. J. J. 2022. Dietary indigestible protein increases post-weaning diarrhoea in piglets irrespective of sanitary housing conditions. Anim. Sci. Proc. 13(2):218–218. 10.1016/j.anscip.2022.03.397

[skaf372-B35] Gozdzik P. , Magkos F., Sledzinski T., Mika A. 2023. Monomethyl branched-chain fatty acids: Health effects and biological mechanisms. Prog. Lipid Res. 90:101226. 10.1016/j.plipres.2023.101226 37094753

[skaf372-B36] Htoo J. K. , Araiza B. A., Sauer W. C., Rademacher M., Zhang Y., Cervantes M., Zijlstra R. T. 2007. Effect of dietary protein content on ileal amino acid digestibility, growth performance, and formation of microbial metabolites in ileal and cecal digesta of early-weaned pigs. J. Anim. Sci. 85(12):3303–3312. 10.2527/jas.2007-0105 17785591

[skaf372-B37] Ighodaro O. M. , Akinloye O. A. 2018. First line defence Antioxidants-Superoxide dismutase (SOD), catalase (CAT) and glutathione peroxidase (GPX): their fundamental role in the entire antioxidant defence grid. Alex. J. Med. 54(4):287–293. 10.1016/j.ajme.2017.09.001

[skaf372-B38] Jiao N. , Wang L., Wang Y., Xu D., Zhang X., Yin J. 2022. Cysteine exerts an essential role in maintaining intestinal integrity and function independent of glutathione. Mol. Nutr. Food Res. 66(3):2100728. 10.1002/mnfr.202100728 34787361

[skaf372-B39] Kennedy M. H. E. , Brosschot T. P., Lawrence K. M., FitzPatrick R. D., Lane J. M., Mariene G. M., Wasmuth J. D., Reynolds L. A. 2021. Small intestinal levels of the branched short-chain fatty acid isovalerate are elevated during infection with Heligmosomoides polygyrus and can promote helminth fecundity. Infect Immun. 89(12):e0022521. 10.1128/IAI.00225-21 34460289 PMC8594610

[skaf372-B40] Kim C. J. , Kovacs-Nolan J., Yang C., Archbold T., Fan M. Z., Mine Y. 2009. L-cysteine supplementation attenuates local inflammation and restores gut homeostasis in a porcine model of colitis. Biochim. Biophys. Acta 1790(10):1161–1169. 10.1016/j.bbagen.2009.05.018 19520150

[skaf372-B41] Kim H. , Shin H., Kim Y. Y. 2023. Effects of different levels of dietary crude protein on growth performance, blood profiles, diarrhea incidence, nutrient digestibility, and odor emission in weaning pigs. Anim. Biosci. 36(8):1228–1240. 10.5713/ab.22.0440 36915927 PMC10330979

[skaf372-B42] Kovács T. , Mikó E., Vida A., Sebő É., Toth J., Csonka T., Boratkó A., Ujlaki G., Lente G., Kovács P. et al 2019. Cadaverine, a metabolite of the microbiome, reduces breast cancer aggressiveness through trace amino acid receptors. Sci. Rep. 9(1):1300. 10.1038/s41598-018-37664-7 30718646 PMC6361949

[skaf372-B43] Kröger S. , Pieper R., Schwelberger H. G., Wang J., Tudela C. V., Aschenbach J. R., Van Kessel A. G., Zentek J. 2013. Diets high in heat-treated soybean meal reduce the histamine-induced epithelial response in the Colon of weaned piglets and increase epithelial catabolism of histamine. PLoS One. 8(11):e80612. 10.1371/journal.pone.0080612 24260435 PMC3833947

[skaf372-B44] Lee E. G. , Yoon Y. C., Yoon J., Lee S. J., Oh Y. K., Kwon S. W. 2021. Systematic review of recent lipidomics approaches toward inflammatory bowel disease. Biomol Ther (Seoul). 29(6):582–595. 10.4062/biomolther.2021.125 34565718 PMC8551739

[skaf372-B45] Li R. , Hou G., Jiang X., Song Z., Fan Z., Hou D. X., He X. 2019a. Different dietary protein sources in low protein diets regulate colonic microbiota and barrier function in a piglet model. Food Funct. 10(10):6417–6428. 10.1039/C9FO01154D\ 31517363

[skaf372-B46] Li R. , Chang L., Hou G., Song Z., Fan Z., He X., Hou D. X. 2019b. Colonic microbiota and metabolites response to different dietary protein sources in a piglet model. Front. Nutr. 6:151. 10.3389/FNUT.2019.00151/BIBTEX 31616670 PMC6768948

[skaf372-B47] Limbach J. R. , Espinosa C. D., Perez-Calvo E., Stein H. H. 2021. Effect of dietary crude protein level on growth performance, blood characteristics, and indicators of intestinal health in weanling pigs. J. Anim. Sci. 99(6):skab166. 10.1093/jas/skab166 34019637 PMC8202089

[skaf372-B48] Lin H. C. , Visek W. J. 1991. Colon mucosal cell damage by ammonia in rats. J. Nutr. 121(6):887–893. 10.1093/jn/121.6.887 2033472

[skaf372-B49] Liu S. , Yang K., Yin J., Chen J., Jiang Q., Wang J., Tan B., Ma X., Liu J. 2025. Effects of dietary protein levels on meat quality, serum antioxidant capacity, and intestinal microorganisms in ningxiang pigs. Antioxidants 14(4):415. 10.3390/antiox14040415 40298662 PMC12024305

[skaf372-B50] Liu Y. , Azad M. A. K., Zhao X., Zhu Q., Kong X. 2022. Dietary crude protein levels alter diarrhea incidence, immunity, and intestinal barrier function of huanjiang mini-pigs during different growth stages. Front. Immunol. 13:908753. 10.3389/fimmu.2022.908753 35874746 PMC9301461

[skaf372-B51] Liu P. P. X. S. , Piao X. S., Thacker P. A., Zeng Z. K., Li P. F., Wang D., Kim S. W. 2010. Chito-oligosaccharide reduces diarrhea incidence and attenuates the immune response of weaned pigs challenged with Escherichia coli K88. J. Anim. Sci. 88(12):3871–3879. 10.2527/jas.2009-2771 20656977

[skaf372-B52] Liu Y. , Azad M. A. K., Zhao X., Zhu Q., Kong X. 2023. Dietary protein levels modulate the antioxidant capacity during different growth stages in Huanjiang mini-pigs. Antioxidants 12(1):148. 10.3390/antiox12010148 36671010 PMC9854851

[skaf372-B53] Long S. , Ma J., Piao X., Li Y., Rasmussen S. H., Liu L. 2021. Enzyme-treated soybean meal enhanced performance via improving immune response, intestinal morphology and barrier function of nursery pigs in antibiotic free diets. Animals (Basel) 11(9):2600. 10.3390/ani11092600 34573566 PMC8471553

[skaf372-B54] Lynegaard J. C. , Kjeldsen N. J., Bache J. K., Weber N. R., Hansen C. F., Nielsen J. P., Amdi C. 2021. Low protein diets without medicinal zinc oxide for weaned pigs reduced diarrhea treatments and average daily gain. Anim. 15(1):100075. 10.1016/j.animal.2020.100075 33516025

[skaf372-B56] McCall I. C. , Betanzos A., Weber D. A., Nava P., Miller G. W., Parkos C. A. 2009. Effects of phenol on barrier function of a human intestinal epithelial cell line correlate with altered tight junction protein localization. Toxicol. Appl. Pharmacol. 241(1):61–70. 10.1016/j.taap.2009.08.002 19679145 PMC2877281

[skaf372-B57] McCracken B. A. , Gaskins H. R., Ruwe-Kaiser P. J., Klasing K. C., Jewell D. E. 1995. Diet-dependent and diet-independent metabolic responses underlie growth stasis of pigs at weaning. J. Nutr. 125(11):2838–2845. 10.1093/jn/125.11.2838 7472664

[skaf372-B58] McCracken B. A. , Spurlock M. E., Roos M. A., Zuckermann F. A., Gaskins H. R. 1999. Weaning anorexia may contribute to local inflammation in the piglet small intestine. J. Nutr. 129(3):613–619. 10.1093/jn/129.3.613 10082764

[skaf372-B59] Newgard C. B. , An J., Bain J. R., Muehlbauer M. J., Stevens R. D., Lien L. F., Haqq A. M., Shah S. H., Arlotto M., Slentz C. A. et al 2009. A branched-chain amino acid-related metabolic signature that differentiates obese and lean humans and contributes to insulin resistance. Cell Metab. 9(4):311–326. 10.1016/j.cmet.2009.02.002 19356713 PMC3640280

[skaf372-B61] Opapeju F. O. , Rademacher M., Blank G., Nyachoti C. M. 2008. Effect of low-protein amino acid-supplemented diets on the growth performance, gut morphology, organ weights and digesta characteristics of weaned pigs. Animal. 2(10):1457–1464. 10.1017/S175173110800270X 22443903

[skaf372-B62] Pearce S.C. , Nisley M.J., Kerr B.J., Sparks C., Gabler N.K. 2024. Effects of dietary protein level on intestinal function and inflammation in nursery pigs. J. Anim. Sci. 102:skae077. 10.1093/jas/skae077 38504643 PMC11015048

[skaf372-B63] Pearce S. C. , Mani V., Boddicker R. L., Johnson J. S., Weber T. E., Ross J. W., Rhoads R. P., Baumgard L. H., Gabler N. K. 2013. Heat stress reduces intestinal barrier integrity and favors intestinal glucose transport in growing pigs. PLoS One. 8(8):e70215. 10.1371/journal.pone.0070215 23936392 PMC3731365

[skaf372-B64] Piel C. , Montagne L., Sève B., Lallès J. P. 2007. Dietary fibre and indigestible protein increase ileal glycoprotein output without impacting colonic crypt goblet cells in weaned piglets. Livest. Sci. 108(1–3):106–108. 10.1016/j.livsci.2007.01.005

[skaf372-B65] Qiu Y. , Liu S., Hou L., Li K., Wang L., Gao K., Yang X., Jiang Z. 2021. Supplemental choline modulates growth performance and gut inflammation by altering the gut microbiota and lipid metabolism in weaned piglets. J. Nutr. 151(1):20–29. 10.1093/jn/nxaa331 33245135

[skaf372-B19399555] Renaud D. L. , Buss L., Wilms J. N., and Steele M. A. 2020. Technical note: is fecal consistency scoring an accurate measure of fecal dry matter in dairy calves?. Journal of Dairy Science. 103(11):10709–10714. 10.3168/jds.2020-18907 32921450

[skaf372-B8895926] Rhine E. D. , Mulvaney R. L., Pratt E. J., and Sims G. K. 1998. Improving the berthelot reaction for determining ammonium in soil extracts and water. Soil Science Soc. of Amer. J. 62(2):473–480. 10.2136/sssaj1998.03615995006200020026x

[skaf372-B67] Rodrigues L. A. , Wellington M. O., González-Vega J. C., Htoo J. K., Van Kessel A. G., Columbus D. A. 2021. Functional amino acid supplementation, regardless of dietary protein content, improves growth performance and immune status of weaned pigs challenged with Salmonella typhimurium. J. Anim. Sci. 99(2):skaa365. 10.1093/jas/skaa365 33529342 PMC8631066

[skaf372-B68] Rodrigues L. A. , Panisson J. C., Van Kessel A. G., Columbus D. A. 2022. Functional amino acid supplementation attenuates the negative effects of plant-based nursery diets on the response of pigs to a subsequent Salmonella typhimurium challenge. J. Anim. Sci. 100(10):skac267. 10.1093/jas/skac267 35976068 PMC9584161

[skaf372-B69] Santoro L. G. , Grant G., Pusztai A. 1999. In vivo degradation and stimulating effect of phaseolin on nitrogen secretion in rats. Plant Foods Hum. Nutr. 53(3):223–236. 10.1023/A:1008025922615 10517281

[skaf372-B70] Silva Y. P. , Bernardi A., Frozza R. L. 2020. The role of short-chain fatty acids from gut microbiota in gut-brain communication. Front. Endocrinol. 11:508738. 10.3389/fendo.2020.00025 PMC7005631 32082260

[skaf372-B71] Sittipo P. , Shim J. W., Lee Y. K. 2019. Microbial metabolites determine host health and the status of some diseases. Int. J. Mol. Sci. 20(21):5296. 10.3390/ijms20215296 31653062 PMC6862038

[skaf372-B72] Sung J.Y. , Johnson T.A., Ragland D., Adeola O. 2023. Impact of ileal indigestible protein on fecal nitrogen excretion and fecal microbiota may be greater compared with total protein concentration of diets in growing pigs. J. Anim. Sci. 101:skac409. 10.1093/jas/skac409 36516453 PMC9890444

[skaf372-B73] Szczesniak O. , Hestad K. A., Hanssen J. F., Rudi K. 2016. Isovaleric acid in stool correlates with human depression. Nutr. Neurosci. 19(7):279–283. 10.1179/1476830515y.0000000007 25710209

[skaf372-B74] van Wettere W. H. E. J. , Willson N. L., Pain S. J., Forder R. E. A. 2016. Effect of oral polyamine supplementation pre-weaning on piglet growth and intestinal characteristics. Animal 10(10):1655–1659. 10.1017/S1751731116000446 26997172

[skaf372-B75] Wang L. , Gao W., Shi H., Hu Q., Lai C. 2022. Effects of replacing fishmeal and soybean protein concentrate with degossypolized cottonseed protein in diets on growth performance, nutrient digestibility, intestinal morphology, cecum microbiome and fermentation of weaned piglets. Animals (Basel) 12(13):1667. 10.3390/ani12131667 35804565 PMC9264811

[skaf372-B76] Wellock I. J. , Fortomaris P. D., Houdijk J. G. M., Wiseman J., Kyriazakis I. 2008. The consequences of non-starch polysaccharide solubility and inclusion level on the health and performance of weaned pigs challenged with enterotoxigenic *Escherichia coli*. Br. J. Nutr. 99(3):520–530. 10.1017/S0007114507819167 17761008

[skaf372-B77] Wen X. , Wang L., Zheng C., Yang X., Ma X., Wu Y., Chen Z., Jiang Z. 2018. Fecal scores and microbial metabolites in weaned piglets fed different protein sources and levels. Anim. Nutr. 4(1):31–36. 10.1016/j.aninu.2017.10.006 30167481 PMC6112360

[skaf372-B78] Windey K. , De Preter V., Verbeke K. 2012. Relevance of protein fermentation to gut health. Mol. Nutr. Food Res. 56(1):184–196. 10.1002/mnfr.201100542 22121108

[skaf372-B79] Xia J. , Fan H., Yang J., Song T., Pang L., Deng H., Ren Z., Deng J. 2022. Research progress on diarrhoea and its mechanism in weaned piglets fed a high‐protein diet. J. Anim. Anim. Physiol. Anim. Nutr. 106(6):1277–1287. 10.1111/jpn.13654 34719816

[skaf372-B80] Xu C. C. , Yang S. F., Zhu L. H., Cai X., Sheng Y. S., Zhu S. W., Xu J. X. 2014. Regulation of N-acetyl cysteine on gut redox status and major microbiota in weaned piglets. J. Anim. Sci. 92(4):1504–1511. 10.2527/jas.2013-6755 24496840

[skaf372-B81] Yang S. J. , Yu X. K., Zuo Q. 2025. Branched-Chain fatty acids and obesity: a narrative review. Nutr. Rev. 83(7):1314–1326, 10.1093/nutrit/nuaf022 40207993

[skaf372-B315436779] Yin L. , Li Jun., Wang M., Wang Q., Li J., Ding N., Yang H., and Yin Y. 2021. Dietary high protein-induced diarrhea and intestinal inflammation by activation of NF-κb signaling in piglets. Animal Nutrition. 7(4):1070–1077. 10.1016/j.aninu.2021.05.002 34738037 PMC8546374

[skaf372-B82] Yun J. H. , Kwon I. K., Lohakare J. D., Choi J. Y., Yong J. S., Zheng J., Cho W. T., Chae B. J. 2005. Comparative efficacy of plant and animal protein sources on the growth performance, nutrient digestibility, morphology and caecal microbiology of early-weaned pigs. Asian-Australasian J. Anim. Sci. 18(9):1285–1293. 10.5713/ajas.2005.1285

[skaf372-B9797758] Zhang H. , Wielen Nvd., Hee Bvd., Wang J., Hendriks W., and ­Gilbert M. 2020. Impact of fermentable protein, by feeding high protein diets, on microbial composition, microbial catabolic activity, gut health and beyond in pigs. Microorganisms. 8(11):1735. 10.3390/microorganisms8111735 33167470 PMC7694525

[skaf372-B84] Zhang S. , Yu Q., Sun Y., Zhang G., Zhang Y., Xin H. 2025. Alleviating the effect of branched-chain fatty acids on the lipopolysaccharide-induced inflammatory response in calf small intestinal epithelial cells. Antioxidants. 14(5):608. 10.3390/antiox14050608 40427489 PMC12109260

[skaf372-B85] Zhao X. , Liu Y., Ding H., Huang P., Yin Y., Deng J., Kong X. 2021. Effects of different dietary protein levels on the growth performance, serum biochemical parameters, fecal nitrogen, and carcass traits of huanjiang mini-pigs. Front. Vet. Sci. 8:777671. 10.3389/fvets.2021.777671 34988141 PMC8720777

